# mRNA transcript quantification in archival samples using multiplexed, color-coded probes

**DOI:** 10.1186/1472-6750-11-46

**Published:** 2011-05-09

**Authors:** Patricia P Reis, Levi Waldron, Rashmi S Goswami, Wei Xu, Yali Xuan, Bayardo Perez-Ordonez, Patrick Gullane, Jonathan Irish, Igor Jurisica, Suzanne Kamel-Reid

**Affiliations:** 1 Division of Applied Molecular Oncology, Princess Margaret Hospital, Ontario Cancer Institute, University Health Network, Toronto, ON, Canada; 2 Dept. of Computer Science, University of Toronto, Toronto, ON, Canada; 3 Ontario Cancer Institute and the Campbell Family Institute for Cancer Research, Toronto, ON, Canada; 4 Dept. of Medical Biophysics, University of Toronto, Toronto, ON, Canada; 5 Dept. of Biostatistics, Princess Margaret Hospital, Ontario Cancer Institute, University Health Network, Toronto, ON, Canada; 6 Dept. of Pathology, Toronto General Hospital, Ontario Cancer Institute, Toronto, ON, Canada; 7 Dept. of Otolaryngology/Surgical Oncology, Princess Margaret Hospital, Ontario Cancer Institute, University Health Network, Toronto, ON, Canada; 8 Dept. of Laboratory Medicine and Pathobiology, University of Toronto, Toronto, ON, Canada

## Abstract

**Background:**

A recently developed probe-based technology, the NanoString nCounter™ gene expression system, has been shown to allow accurate mRNA transcript quantification using low amounts of total RNA. We assessed the ability of this technology for mRNA expression quantification in archived formalin-fixed, paraffin-embedded (FFPE) oral carcinoma samples.

**Results:**

We measured the mRNA transcript abundance of 20 genes (*COL3A1*, *COL4A1*, *COL5A1*, *COL5A2*, *CTHRC1*, *CXCL1*, *CXCL13*, *MMP1*, *P4HA2*, *PDPN*, *PLOD2*, *POSTN*, *SDHA*, *SERPINE1*, *SERPINE2*, *SERPINH1*, *THBS2*, *TNC*, *GAPDH*, *RPS18*) in 38 samples (19 paired fresh-frozen and FFPE oral carcinoma tissues, archived from 1997-2008) by both NanoString and SYBR Green I fluorescent dye-based quantitative real-time PCR (RQ-PCR). We compared gene expression data obtained by NanoString *vs*. RQ-PCR in both fresh-frozen and FFPE samples. Fresh-frozen samples showed a good overall Pearson correlation of 0.78, and FFPE samples showed a lower overall correlation coefficient of 0.59, which is likely due to sample quality. We found a higher correlation coefficient between fresh-frozen and FFPE samples analyzed by NanoString (r = 0.90) compared to fresh-frozen and FFPE samples analyzed by RQ-PCR (r = 0.50). In addition, NanoString data showed a higher mean correlation (r = 0.94) between individual fresh-frozen and FFPE sample pairs compared to RQ-PCR (r = 0.53).

**Conclusions:**

Based on our results, we conclude that both technologies are useful for gene expression quantification in fresh-frozen or FFPE tissues; however, the probe-based NanoString method achieved superior gene expression quantification results when compared to RQ-PCR in archived FFPE samples. We believe that this newly developed technique is optimal for large-scale validation studies using total RNA isolated from archived, FFPE samples.

## Background

A vast collection of formalin-fixed and paraffin-embedded (FFPE) tissue samples are currently archived in anatomical pathology laboratories and tissue banks around the world. These samples are an extremely valuable source for molecular biology studies, since they have been annotated with varied information on disease states and patient follow-up, such as disease progression in cancer and prognosis/survival data. Although FFPE samples provide an ample source for genetic studies, formalin fixation is known to affect the quality of DNA and RNA extracted from FFPE samples and its downstream applications, such as amplification by the Polymerase Chain Reaction (PCR) or microarrays [1].

Von Ahlfen *et al*., 2007 [1] described the different factors (e.g. fixation, storage time and conditions) that can influence the integrity of RNA extracted from FFPE tissues, and its downstream applications. They showed that differences in storage time and temperature had a large effect on the degree of RNA degradation. In their study, RNA samples extracted within 1 to 3 days after formalin fixation and paraffin embedding maintained their integrity. Similarly, RNA isolated from FFPE samples that were stored at 4°C showed higher quality compared to samples stored at room temperature or at 37°C. They also reported that RNA fragmentation occurs gradually over time. It is also known that cDNA synthesis from FFPE-derived RNA is limited due to the use of formaldehyde during fixation. Formaldehyde induces chemical modification of RNA, characterized by the formation of methylene crosslinks between nucleic acids and protein. These chemical modifications can be partially irreversible [2], limiting the application of techniques such as reverse transcription, which uses mRNA as a template for cDNA synthesis. A fixation time over 24 hours was shown to result in a higher number of irreversible crosslinks [3,4]. Overall, fixation time and method of RNA extraction are the main factors that determine the extent of methylene crosslinks [1].

A recently developed probe-based technology, the NanoString nCounter™ gene expression system, has been shown to allow accurate mRNA expression quantification using low amounts of total RNA [5]. This technique is based on direct measurement of transcript abundance, by using multiplexed, color-coded probe pairs, and is able to detect as little as 0.5 fM of mRNA transcripts; described in detail in Geiss *et al.*, 2008 [5]. In brief, unique pairs of a capture and a reporter probe are synthesized for each gene of interest, allowing ~800 genes to be multiplexed, and their mRNA transcript levels measured, in a single experiment, for each sample. In addition, in a recent study, mRNA expression levels obtained using NanoString were more sensitive than microarrays and yielded similar sensitivity when compared to two quantitative real-time PCR techniques: TaqMan-based RQ-PCR and SYBR Green I fluorescent dye-based RQ-PCR [5]. Although NanoString and RQ-PCR were shown to produce comparable data in good quality samples, NanoString is hybridization-based, and does not require reverse transcription of mRNA and subsequent cDNA amplification. This feature of NanoString technology offers advantages over PCR-based methods, including the absence of amplification bias, which may be higher when using fragmented RNA isolated from FFPE specimens. In addition, NanoString assays do not require the use of control samples, since absolute transcript abundance is determined for each single sample and normalized against the expression of housekeeping genes in that same sample [5].

Although NanoString technology has been optimized for gene expression analysis using formalin-fixed samples, to our knowledge we are the first to report the use of this technology for mRNA transcript quantification using clinical, archival, FFPE cancer tissues. In our pilot study, we used the NanoString nCounter™ assay for gene expression analysis of archival oral carcinoma samples. In order to show that mRNA levels obtained by NanoString analysis of FFPE tissues were accurate, we compared quantification data obtained using RNA isolated from paired fresh-frozen and FFPE oral cancer samples. Our goal was to determine whether this technology could be applied for accurate gene expression quantification using archived, FFPE oral cancer tissues. We also aimed to compare whether quantification data obtained by NanoString achieved a higher correlation than data obtained by SYBR Green I fluorescent dye-based RQ-PCR, using the same paired fresh-frozen and FFPE samples.

## Methods

### Tissue samples

This study was performed under approval of the Research Ethics Board at University Health Network. Tissues were collected with informed patient consent. Study samples included primary fresh-frozen and formalin-fixed, paraffin-embedded (FFPE) tumor samples from 19 patients with oral squamous cell carcinoma. All patients had surgery as primary treatment. Fresh-frozen tissues were collected at the time of surgical resection, and samples were snap frozen and kept in liquid nitrogen until RNA extraction. RNA from these tumor samples was extracted and kept at -80C for long term storage. Representative FFPE tissue sections were obtained from the same tumor samples. We collected a total of 38 tumor samples (paired fresh-frozen and FFPE) from 19 patients. In addition, we included the analysis of a commercially available human universal RNA (pool of cancer cell lines) (Stratagene) and human normal tongue RNA (Stratagene); these samples were used as quality controls, since they are a source of high quality RNA, and have been previously used in other studies [6,7].

### RNA extraction and cDNA synthesis

Total RNA was isolated from fresh-frozen tissues using Trizol reagent (Life Technologies, Inc., Burlington, ON, Canada), followed by purification using the Qiagen RNeasy kit and treatment with the DNase RNase-free set (Qiagen, Valencia, CA, USA). RNA extraction and purification steps were performed according to the manufacturers' instructions.

For FFPE tissues, one tissue section was taken from each specimen, prior to RNA extraction, stained with hematoxylin and eosin (H&E) and examined by a pathologist (B.P-O), to ensure that tissues contained >80% tumor cells. RNA was isolated from five 10 μm sections from FFPE samples, using the RecoverAll™ Total Nucleic Acid Isolation Kit (Ambion, Austin, TX, USA), following the manufacturer's procedures. RNA extracted from both fresh-frozen and FFPE tissues was assessed for quantity using Nanodrop 1000 (Nanodrop), and for quality using the 2100 Bioanalyzer (Agilent Technologies, Canada).

For RQ-PCR experiments, cDNA was synthesized from 1 μg total RNA isolated from fresh-frozen or FFPE tissues, using the M-MLV reverse transcriptase enzyme and according to manufacturer's protocol (Invitrogen).

### Gene expression quantification using multiplexed, color-coded probe pairs (NanoString nCounter™)

Genes selected for testing in this technical report are frequently over-expressed in oral cancer (our own data, currently submitted for publication elsewhere). Probe sets for each gene were designed and synthesized by NanoString nCounter™ technologies (Table 1). Probe sets of 100 bp in length were designed to hybridize specifically to each mRNA target. Probes contained one capture probe linked to biotin and one reporter probe attached to a color-coded molecular tag, according to the nCounter™ code-set design.

**Table 1:** Probe sets for genes of interest used for Nanostring analysis

Gene Symbol	Accession Number	Target Region	Target Sequence
*COL3A1*	NM_000090.3	180-280	TTGGCACAACAGGAAGCTGTTGAAGGAGGATGTTCCCATCTTGGTCAGTCCTATGCGGATAGAGATGTCTGGAAGCCAGAACCATGCCAAATATGTGTCT

*COL4A1*	NM_001845.4	780-880	TGGGCTTAAGTTTTCAAGGACCAAAAGGTGACAAGGGTGACCAAGGGGTCAGTGGGCCTCCAGGAGTAβCCAGGACAAGCTCAAGTTCAAGAAAAAGGAGA

*COL5A1*	NM_000093.3	6345-6445	GTAAAGGTCATCCCACCATCACCAAAGCCTCCGTTTTTAACAACCTCCAACACGATCCATTTAGAGGCCAAATGTCATTCTGCAGGTGCCTTCCCGATGG

*COL5A2*	NM_000393.3	4075-4175	GGTTCATGCTACCCTGAAGTCACTCAGTAGTCAGATTGAAACCATGCGCAGCCCCGATGGCTCGAAAAAGCACCCAGCCCGCACGTGTGATGACCTAAAG

*CTHRC1*	NM_138455.2	685-785	CTGTGGAAGGACTTTGTGAAGGAATTGGTGCTGGATTAGTGGATGTTGCTATCTGGGTTGGCACTTGTTCAGATTACCCAAAAGGAGATGCTTCTACTGG

*CXCL1*	NM_001511.1	445-545	AGGCCCTGCCCTTATAGGAACAGAAGAGGAAAGAGAGACACAGCTGCAGAGGCCACCTGGATTGTGCCTAATGTGTTTGAGCATCGCTTAGGAGAAGTCT

*CXCL13*	NM_006419.2	0-100	GAGAAGATGTTTGAAAAAACTGACTCTGCTAATGAGCCTGGACTCAGAGCTCAAGTCTGAACTCTACCTCCAGACAGAATGAAGTTCATCTCGACATCTC

*MMP1*	NM_002421.3	1117-1217	AAATGGGCTTGAAGCTGCTTACGAATTTGCCGACAGAGATGAAGTCCGGTTTTTCAAAGGGAATAAGTACTGGGCTGTTCAGGGACAGAATGTGCTACAC

*P4HA2*	NM_001017974.1	1600-1700	TGTGCTTGTGGGCTGCAAGTGGGTCTCCAATAAGTGGTTCCATGAACGAGGACAGGAGTTCTTGAGACCTTGTGGATCAACAGAAGTTGACTGACATCCT

*PDPN*	NM_006474.4	431-531	CTCCAGGAACCAGCGAAGACCGCTATAAGTCTGGCTTGACAACTCTGGTGGCAACAAGTGTCAACAGTGTAACAGGCATTCGCATCGAGGATCTGCCAAC

*PLOD2*	NM_182943.2	2590-2690	AAACATTGCACTTAATAACGTGGGAGAAGACTTTCAGGGAGGTGGTTGCAAATTTCTAAGGTACAATTGCTCTATTGAGTCACCACGAAAAGGCTGGAGC

*POSTN*	NM_001135935.1	910-1010	AGAGACGGTCACTTCACACTCTTTGCTCCCACCAATGAGGCTTTTGAGAAACTTCCACGAGGTGTCCTAGAAAGGATCATGGGAGACAAAGTGGCTTCCG

*SDHA*	NM_004168.1	230-330	TGGAGGGGCAGGCTTGCGAGCTGCATTTGGCCTTTCTGAGGCAGGGTTTAATACAGCATGTGTTACCAAGCTGTTTCCTACCAGGTCACACACTGTTGCA

*SERPINE1*	NM_000602.2	2470-2570	TGTGTTCAATAGATTTAGGAGCAGAAATGCAAGGGGCTGCATGACCTACCAGGACAGAACTTTCCCCAATTACAGGGTGACTCACAGCCGCATTGGTGAC

*SERPINE2*	NM_006216.2	240-340	CGCTGCCTTCCATCTGCTCCCACTTCAATCCTCTGTCTCTCGAGGAACTAGGCTCCAACACGGGGATCCAGGTTTTCAATCAGATTGTGAAGTCGAGGCC

*SERPINH1*	NM_001235.2	880-980	ATGGTGGACAACCGTGGCTTCATGGTGACTCGGTCCTATACCGTGGGTGTCATGATGATGCACCGGACAGGCCTCTACAACTACTACGACGACGAGAAGG

*THBS2*	NM_003247.2	4460-4560	AAACATCCTTGCAAATGGGTGTGACGCGGTTCCAGATGTGGATTTGGCAAAACCTCATTTAAGTAAAAGGTTAGCAGAGCAAAGTGCGGTGCTTTAGCTG

*TNC*	NM_002160.1	6885-6985	CAGAAATCTTGAAGGCAGGCGCAAACGGGCATAAATTGGAGGGACCACTGGGTGAGAGAGGAATAAGGCGGCCCAGAGCGAGGAAAGGATTTTACCAAAG

*GAPDH*	NM_002046.3	35-135	TCCTCCTGTTCGACAGTCAGCCGCATCTTCTTTTGCGTCGCCAGCCGAGCCACATCGCTCAGACACCATGGGGAAGGTGAAGGTCGGAGTCAACGGATTT

*RPS18*	NM_022551.2	110-210	GCGGCGGAAAATAGCCTTTGCCATCACTGCCATTAAGGGTGTGGGCCGAAGATATGCTCATGTGGTGTTGAGGAAAGCAGACATTGACCTCACCAAGAGG

*GAPDH *and *RPS18 *were used as internal controls for normalization of Nanostring data.

RNA samples were randomized using a numerical ID, in order to blind samples for sample type (fresh-frozen or FFPE) and sample pairs. Samples were then subjected to NanoString nCounter™ analysis by the University Health Network Microarray Centre (http://www.microarrays.ca/) at the Medical Discovery District (MaRS), Toronto, ON, Canada. The detailed protocol for mRNA transcript quantification analysis, including sample preparation, hybridization, detection and scanning followed the manufacturer's recommendations, and are available at http://www.nanostring.com/uploads/Manual_Gene_Expression_Assay.pdf/ under http://www.nanostring.com/applications/subpage.asp?id=343. We used 100 ng of total RNA isolated from fresh-frozen tissues, as suggested by the manufacturer. FFPE tissues required a higher amount of total RNA (400 ng) for detection of probe signals. Technical replicates of three paired fresh-frozen and FFPE tissues were included. Data were analyzed using the nCounter™ digital analyzer software, available at http://www.nanostring.com/support/ncounter/.

### Quantitative real-time RT-PCR

In addition, we performed RQ-PCR analysis in the same fresh-frozen and FFPE samples and compared this to gene expression data determined by the NanoString nCounter assay. RQ-PCR analysis was performed as previously described, using SYBR Green I fluorescent dye [8,9]. Gene IDs and primer sequences are described in Table 2. Primer sequences were designed using Primer-BLAST (http://www.ncbi.nlm.nih.gov/tools/primer-blast/). Gene expression levels were normalized against the average Ct (cycle threshold) values for the two internal control genes (*GAPDH *and *RPS18*) and calculated relative to a commercially available normal tongue reference RNA (Stratagene). Ct values were extracted using the SDS 2.3 software (Applied Biosystems). Data analysis was performed using the ΔΔCt method [10].

**Table 2:** Primer sequences used in the RQ-PCR experiments

Gene symbol	Primer sequence	Amplicon length
*GAPDH*	Forward 5'-CCTGTTCGACAGTCAGCCGCAT-3' Reverse 5'-GACTCCGACCTTCACCTTCCCC-3'	87 bp

*RPS18*	Forward 5'-GCGGCGGAAAATAGCCTTTGCC-3' Reverse 5'-CCTCTTGGTGAGGTCAATGTCTGC-3'	100 bp

*MMP1*	Forward 5'-CAAATGGGCTTGAAGCTGCTTACG-3' Reverse 5'-GTGTAGCACATTCTGTCCCTGAACA-3'	101 bp

*COL4A1*	Forward 5'-AAGGACCAAAAGGTGACAAGGGTGA-3' Reverse 5'-GAACTTGAGCTTGTCCTGGTACTCC-3'	72 bp

*COL5A1*	Forward 5'-GTCATCCCACCATCACCAAAGCC-3' Reverse 5'-ATCGGGAAGGCACCTGCAGAATG-3'	92 bp

*THBS2*	Forward 5'-TTGCAAATGGGTGTGACGCGGT-3' Reverse 5'-AAGCACCGCACTTTGCTCTGCT-3'	86 bp

*TNC*	Forward 5'-ACGAACACTCAATCCAGTTTGCTGA-3' Reverse 5'-TGGAATTTATGCCCGTTTGCGCC-3'	89 bp

*COL3A1*	Forward 5'-TGGCACAACAGGAAGCTGTTGAAGG-3' Reverse 5'-ACACATATTTGGCATGGTTCTGGCT-3'	97 bp

*COL5A2*	Forward 5'-TCATGCTACCCTGAAGTCACTCAGT-3' Reverse 5'-AGGTCATCACACGTGCGGGC-3'	93 bp

*PDPN*	Forward 5'-CAGGAACCAGCGAAGACCGCT-3' Reverse 5'-TGGCAGATCCTCGATGCGAATGC-3'	95 bp

*POSTN*	Forward 5'-CGGTCACTTCACACTCTTTGCTCCC-3' Reverse 5'-CGGAAGCCACTTTGTCTCCCATGA-3'	95 bp

*SERPINE2*	Forward 5'-ACCATGAACTGGCATCTCCCCCT-3' Reverse 5'-TGGAGCCTAGTTCCTCGAGAGACA-3'	100 bp

*SERPINH1*	Forward 5'-CCGTGGCTTCATGGTGACTCGG-3' Reverse 5'-AGTAGTTGTAGAGGCCTGTCCGGT-3'	74 bp

*SDHA*	Forward 5'-CTCCAAGCCCATCCAGGGGCAA-3' Reverse 5'-CAGAGTGACCTTCCCAGTGCCAA-3'	100 bp

*PLOD2*	Forward 5'-TGGCTCTTTGCCGAAATGCTAGAG-3' Reverse 5'-GGGGGCTGAGCATTTGGAATGTTT-3'	87 bp

*P4HA2*	Forward: 5'-AGGAGCTGCCAAAGCCCTGA-3' Reverse: 5'-ACCTGCTCCATCCACAACACCG-3'	170 bp

*CTHRC1*	Forward: 5'-TTGTTCAGTGGCTCACTTCG-3' Reverse: 5'-TTCAATGGGAAGAGGTCCTG-3'	102 bp

*CXCL1*	Forward: 5'-ATTTCTGAGGAGCCTGCAAC-3' Reverse: 5'-CACATACATTCCCCTGCCTT-3'	100 bp

*CXCL13*	Forward: 5'-GAGCCTGTCAAGAGGCAAAG-3' Reverse: 5'-CTGGGGATCTTCGAATGCTA-3'	142 bp

*SERPINE1 *was excluded from RQ-PCR analysis since no primer pairs tested showed good efficiency for amplification in FFPE samples.

Primer sequences used yielded short amplicon lengths, as indicated.

### Statistical analysis

Absolute mRNA quantification values obtained by NanoString as well as relative expression values obtained by RQ-PCR were log2-transformed. Summary statistics as median, mean, range were provided. Pair-wise Pearson product-moment correlation analysis [11] was applied to test the correlation between gene expression data obtained by NanoString and RQ-PCR analysis in fresh-frozen *vs*. FFPE samples, as well as the correlation between NanoString and RQ-PCR data in fresh-frozen or FFPE samples. Both overall correlation and correlation across sample pairs were calculated. Statistical analyses were performed using version 9.2 of the SAS system and user's guide (SAS Institute, Cary, NC). In addition, Pearson correlation between sample pairs was plotted as heatmaps, in order to visualize the grouping of similar samples. Heatmaps were generated by hierarchical clustering analysis, using *hclust *R function, in R statistical environment [12].

## Results

### Technical data on sample quality

**Figure 1:**
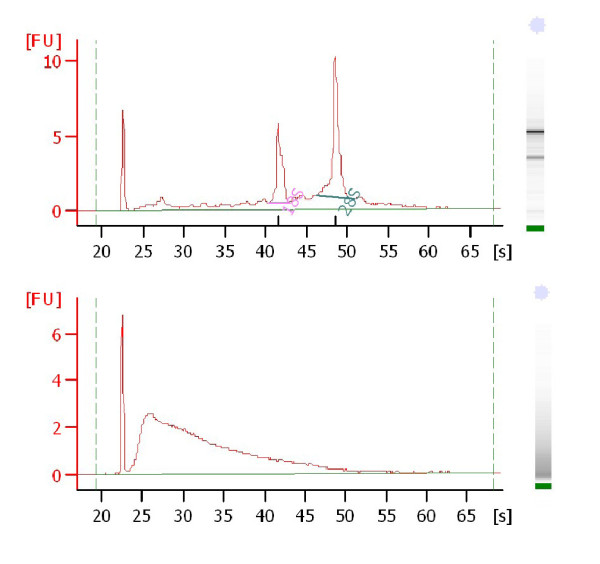
**Bioanalyzer assessment of RNA integrity**. Representative examples of RNA integrity results after Bioanalyzer assessment of paired fresh-frozen (upper) and FFPE (lower) samples. The fresh-frozen sample shown in the upper panel had a RIN = 8.7 and the FFPE sample shown in the lower panel had a RIN = 2.3.

Bioanalyzer results for fresh-frozen samples showed a mean RNA integrity number (RIN) of 8.3 (range 4.6-9.8), with the majority of fresh-frozen samples (13/19) having a RIN ≥8. FFPE samples were degraded and the mean RIN was 2.3 (range 1.5-2.5); this result was expected since FFPE samples are archival tissues. Representative examples of the Bioanalyzer results for one fresh-frozen and one FFPE sample are shown in Figure 1. FFPE samples used in our study have been archived from a time period between 1997-2008.

### Correlation between mRNA transcript quantification in fresh-frozen vs. FFPE samples (NanoString)

**Figure 2:**
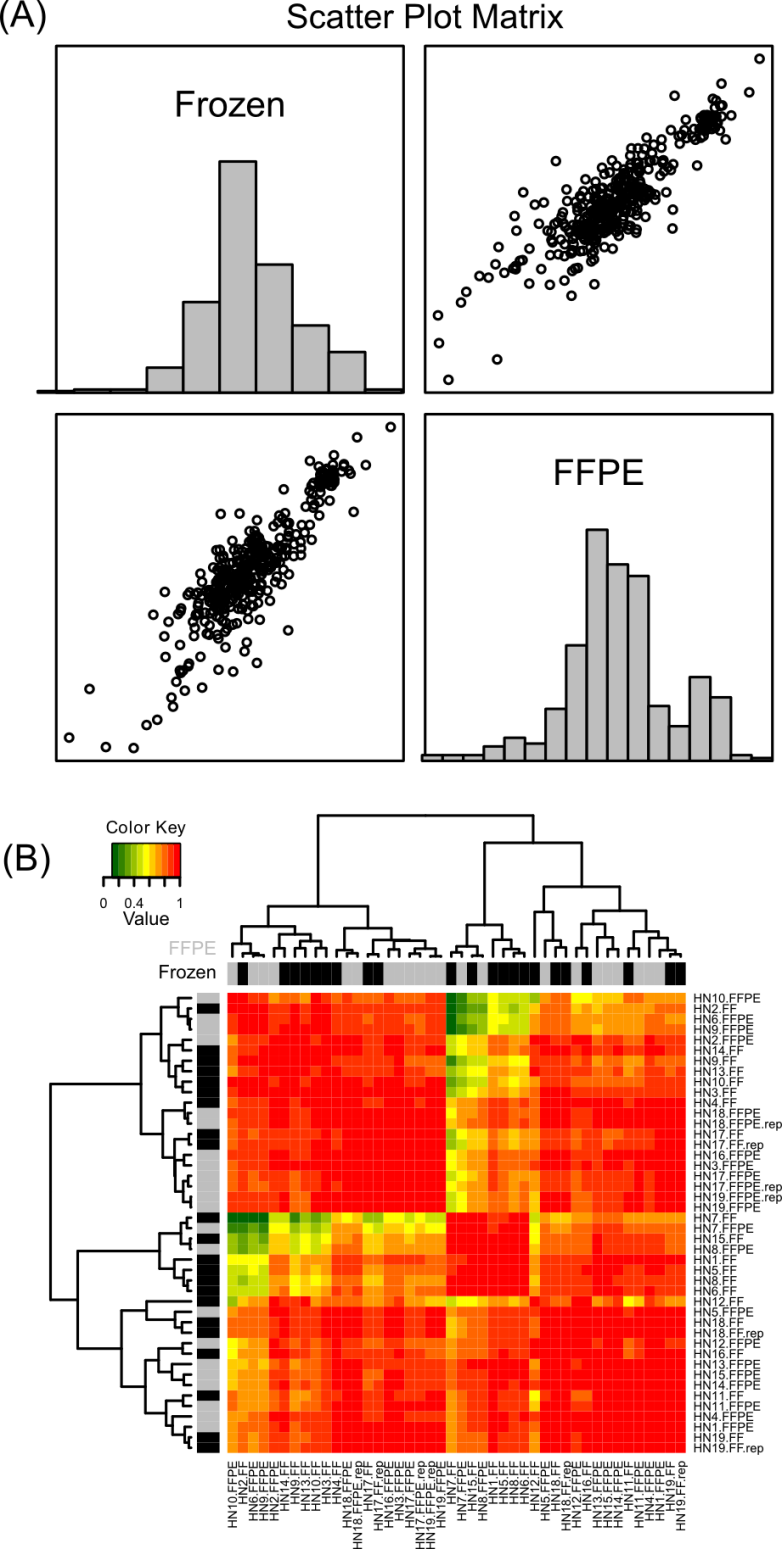
**Correlation of results obtained from Nanostring analysis of paired fresh-frozen and FFPE tissues**. Scatter plot matrix (left panel, A) for the normalized mRNA transcript quantification values obtained by Nanostring analysis of 19 fresh-frozen *vs*. FFPE sample pairs (n = 38 samples). In this analysis, the pair-wise Pearson product-moment correlation coefficient was 0.90 (p < 0.0001). The right panel (B) shows a heatmap analysis for the Pearson correlation of absolute mRNA transcript abundance as determined by Nanostring, for all pair-wise combinations of samples. These results show a good-high correlation between absolute mRNA transcript quantification data in fresh-frozen *vs*. FFPE tissues using Nanostring analysis. Fresh-frozen and FFPE tissues are interspersed, and all technical replicates are adjacent in all cases. Gene expression patterns are highly consistent among the large majority of samples.

Raw data quantification values obtained by NanoString were log2 transformed, and values derived from the 19 paired fresh-frozen and FFPE samples were compared. The pair-wise Pearson product-moment correlation was 0.90 (p < 0.0001). The scatter plot and histogram for log2 values from fresh-frozen and FFPE samples are shown in Figure 2A. Analysis of the three replicate pairs (log2 transformed values) demonstrated a correlation of 0.93 (p < 0.0001). In addition, we performed unsupervised hierarchical clustering analysis of these data, and heatmaps are shown in Figure 2B.

We also performed a correlation analysis between mRNA transcript quantification values (log2 transformed values) for each pair of fresh-frozen *versus *FFPE sample (sample by sample comparison). This analysis is important as it allows us to determine whether the amount of mRNA transcripts of a given gene is maintained in individual sample pairs. The mean correlation coefficient obtained was 0.94, with a minimum correlation of 0.77 and a maximum correlation of 0.99.

### Correlation between gene expression levels in fresh-frozen vs. FFPE samples (RQ-PCR)

**Figure 3:**
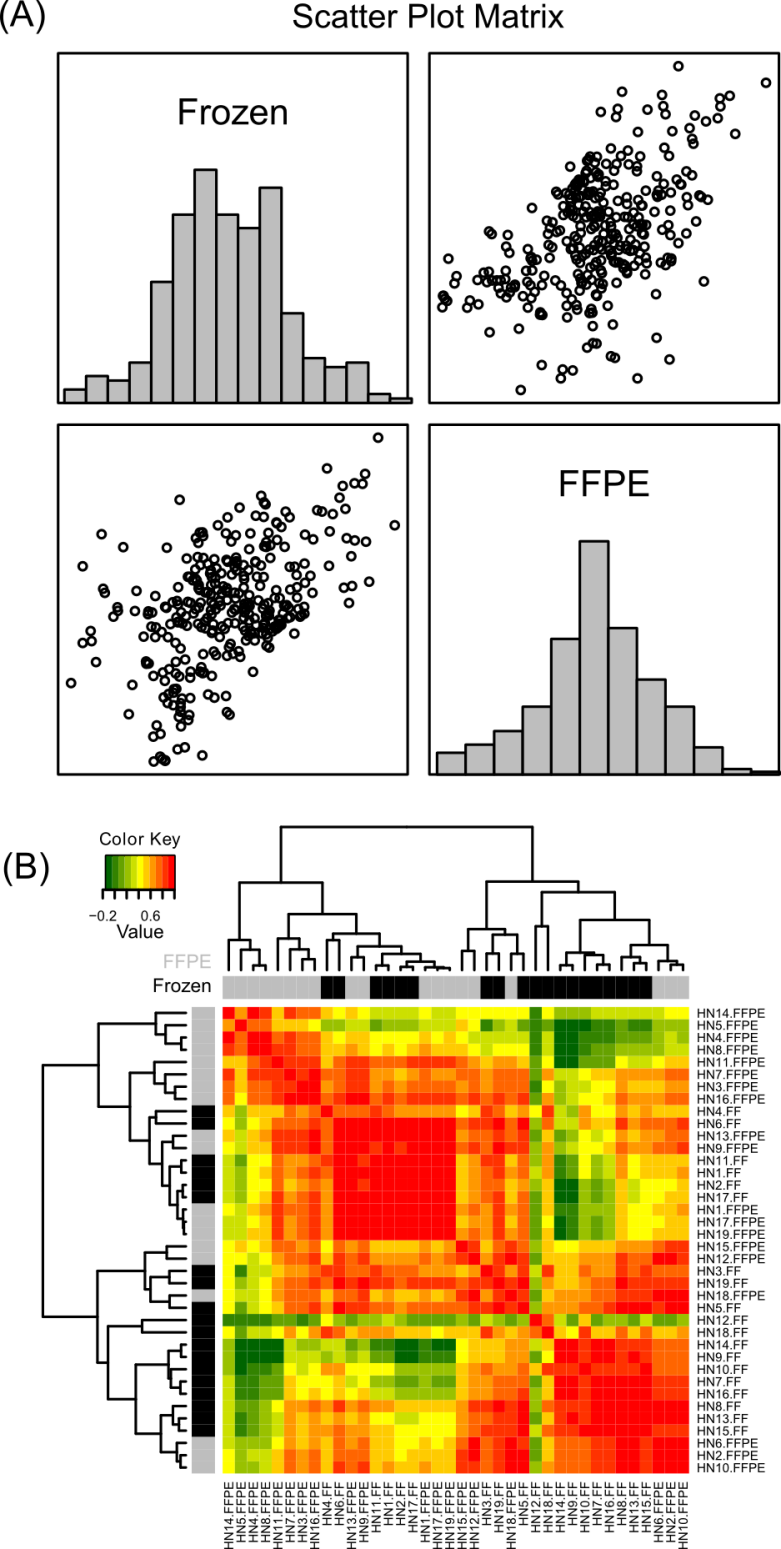
**Correlation of results obtained from RQ-PCR analysis of paired fresh-frozen and FFPE tissues**. Scatter plot matrix (left panel, A) showing normalized gene expression data obtained by RQ-PCR analysis of the 19 fresh-frozen *vs*. FFPE sample pairs (n = 38 samples). The pair-wise Pearson product-moment correlation coefficient was 0.50 (p < 0.0001). The right panel (B) shows a heatmap analysis for the Pearson correlation of gene expression abundance as determined by RQ-PCR, for all pair-wise combinations of samples. A low-moderate correlation is observed between mRNA transcript quantification data in fresh-frozen *vs*. FFPE tissues, and tissues tend to cluster according to storage method.

We also compared gene expression levels determined by RQ-PCR analysis in fresh-frozen *versus *FFPE samples. The overall pair-wise Pearson product-moment correlation coefficient was 0.53 (p < 0.0001) (Figure 3A). Heatmap analysis of these data is shown in Figure 3B. A sample-by-sample (fresh-frozen/FFPE sample pair) correlation analysis of RQ-PCR data revealed a mean correlation of 0.54, varying between 0.12 and 0.99, with the majority of sample pairs (12/19) showing a correlation ≥0.50.

### Comparison of mRNA quantification data using NanoString versus RQ-PCR

Since all RNA samples isolated from FFPE tissues were degraded, as confirmed by Bioanalyzer analysis, we expected that a probe-based assay would generate more accurate gene expression quantification data compared to amplification-based assays, such as RQ-PCR.

**Figure 4:**
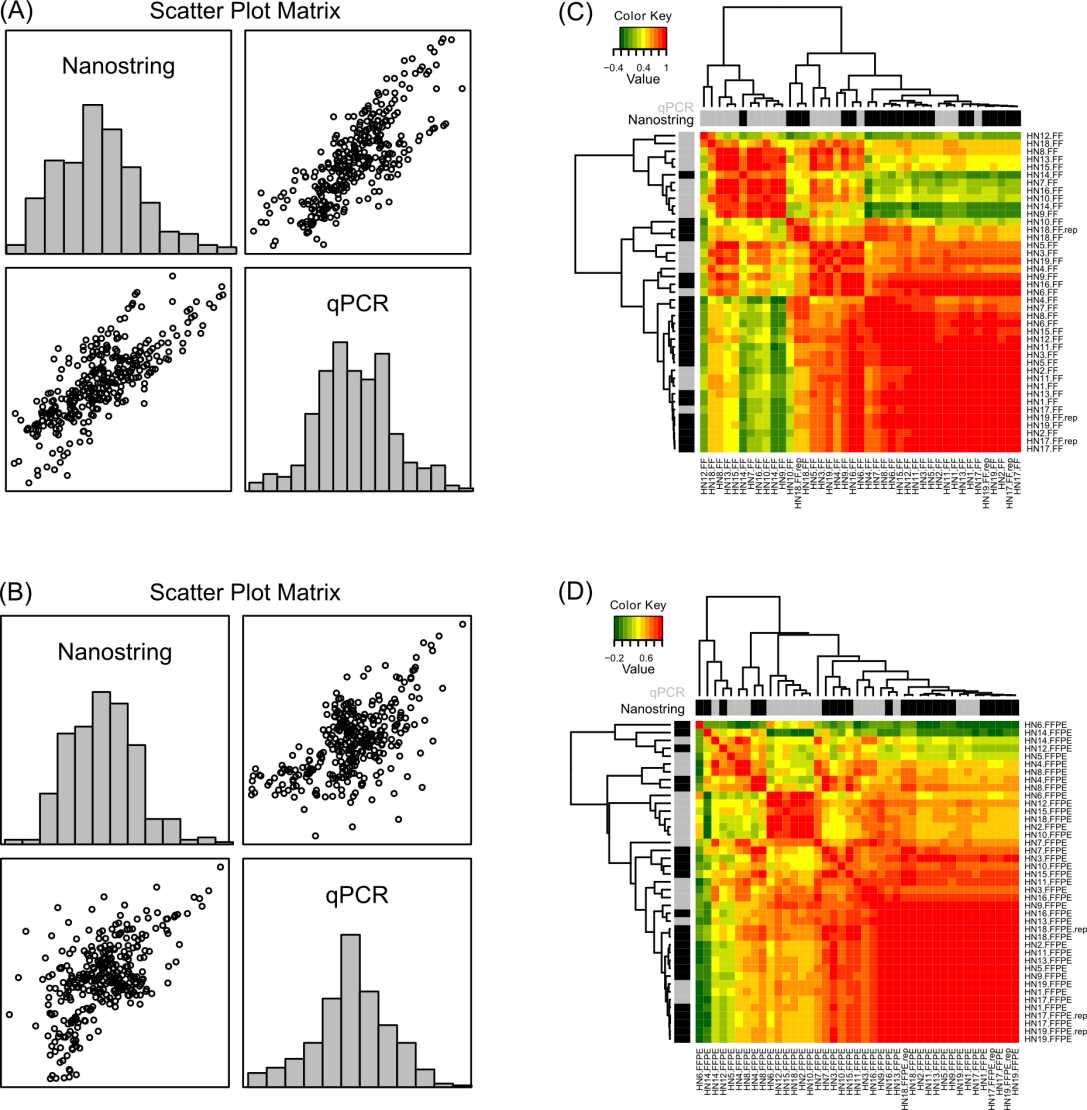
**Correlation between data obtained from Nanostring and RQ-PCR analysis on fresh-frozen and FFPE tissues**. Scatter-plot matrices examining the correlation between Nanostring and RQ-PCR data in fresh-frozen (A) and FFPE (B) samples. Scatter plot matrices show normalized quantification values. The pair-wise Pearson product-moment correlation coefficient for Nanostring *vs*. RQ-PCR data in fresh-frozen samples was r = 0.78 (*p < 0.0001*); this same analysis revealed a lower correlation coefficient in FFPE samples (r = 0.59) (*p < 0.0001*). A corresponding heatmap for the Pearson correlation of gene expression abundance in fresh-frozen (FF) and FFPE samples using Nanostring *vs*. RQ-PCR is shown to the right of each scatter plot (C and D respectively). These results show a good correlation between Nanostring and RQ-PCR in fresh-frozen samples, and a lower correlation between data obtained using these two different technologies, when using clinical, archival, FFPE tissues.

For each sample type (fresh-frozen or FFPE), we compared mRNA transcript quantification as determined by NanoString analysis and gene expression levels as determined by RQ-PCR. For fresh-frozen tissues, this comparison analysis showed that the overall pair-wise Pearson product-moment correlation coefficient was 0.78 (p < 0.0001). Figure 4A shows the scatter plot for the Log(NanoString) *vs*. Log(QPCR) and their histogram in fresh-frozen tissues. This same analysis in FFPE samples showed a lower overall correlation coefficient of 0.59 (p < 0.0001); 11/19 FFPE sample pairs showed a correlation ≥0.60. Figure 4B shows the scatter plot for the Log(NanoString) *vs*. Log(QPCR) and their histogram in FFPE tissues. Unsupervised hierarchical clustering analysis of these data was performed and corresponding heatmaps are shown in Figure 4C and 4D.

## Discussion

In this pilot study, we showed that NanoString technology is suitable for accurately detecting and measuring mRNA transcript levels in clinical, archival, FFPE oral carcinoma samples. Our results demonstrated that this probe-based assay (NanoString) achieved a good overall Pearson correlation when we compared mRNA transcript quantification results between paired fresh-frozen and FFPE samples. In addition, correlation coefficients were determined in a sample-by-sample comparison, and results showed that mRNA levels in single sample pairs (fresh-frozen and FFPE) were maintained across the sample pairs when using NanoString technology. When we compared gene expression levels obtained by RQ-PCR, we obtained a lower overall correlation coefficient between fresh-frozen and FFPE tissues, and across sample pairs. These results suggest that mRNA transcript levels are more concordant between fresh-frozen and FFPE sample pairs when using NanoString technology.

A recently published study [13] evaluated the performance of quantitative real-time PCR using TaqMan assays (TaqMan Low Density Arrays platform), for gene expression analysis using paired fresh-frozen and FFPE breast cancer samples. The investigators found a good overall correlation coefficient of 0.81 between fresh-frozen and FFPE samples; however, when they compared individual sample pairs, they found a low correlation of 0.33, with variability of 0.005-0.81. These authors suggested that the extensive RNA sample degradation in FFPE samples is likely the cause for the low correlation coefficients observed across sample pairs [13]. Indeed, Bioanalyzer results for our samples showed that fresh-frozen tissues had a good quality RIN and were suitable for gene expression analysis, while FFPE tissues were degraded and had a low RNA integrity number. This RNA degradation in FFPE samples also resulted in higher Ct values initially detectable by RQ-PCR, with loss of amplifiable templates. The low RIN characteristic of FFPE samples did not seem to have an effect on the efficiency of NanoString results, however, when we compared quantification values obtained using RNA isolated from fresh-frozen *vs*. FFPE tissues.

Although quantitative PCR-based assays have been used for gene expression analysis in FFPE samples [13-15], these assays do carry some disadvantages, such as the need for optimization strategies aimed at reducing amplification bias and increasing the number of detectable amplicons when using RNA extracted from FFPE samples. To date, some of the recommended strategies include optimization of the RNA extraction method and designing primers able to detect short amplicons [16]. In our study, primers for RQ-PCR experiments yielded amplicon lengths between 72-170 bp (as detailed in Table 2). Only 2/19 primer pairs yield amplicons >110 bp in size. Such short amplicons are well-suited for PCR amplification using FFPE samples. Our results showed that, although we did obtain gene expression data using RQ-PCR in our FFPE samples, both the overall and the sample-by-sample correlation between fresh-frozen and FFPE samples was notably lower for RQ-PCR data than for data obtained using NanoString. This suggests that this newly developed technology, NanoString nCounter™, offers advantages over RQ-PCR for gene expression analysis in archival FFPE samples.

## Conclusions

We found that the multiplexed, color-coded probe-based method (NanoString nCounter™) achieved superior gene expression quantification results when compared to RQ-PCR, when using total RNA extracted from clinical, archival, FFPE samples. Such technology could thus be very useful for applications requiring the use of clinical archival material, such as large scale validation of gene expression data generated by microarrays for generation of tissue specific gene expression signatures.

## List of abbreviations

Ct: cycle threshold; FFPE: formalin fixed, paraffin embedded; H&E: hematoxylin and eosin; M-MLV RT enzyme: Moloney Murine Leukemia Virus reverse transcriptase enzyme; PCR: polymerase chain reaction; RIN: RNA integrity number; RQ-PCR: Quantitative real-time PCR; SAS: Statistical analysis system; SDS: Sequence Detection System;

## Authors' contributions

PPR, RSG and SKR designed the study. LW and WX performed statistical data analyses. PPR wrote the manuscript. PPR and RSG performed data interpretation. YX performed quantitative PCR assays. RSG, LW, WX, IJ and SKR edited the manuscript. BPO performed histopathological analyses and evaluated all samples. PG and JI provided samples and reviewed the manuscript. All authors read and approved the final manuscript.
